# Operational impact of decreased turnaround times for *Candida auris* screening tests in a tertiary academic medical center

**DOI:** 10.1017/ash.2023.445

**Published:** 2023-10-18

**Authors:** Sebastian Arenas, Samira Patel, Spencer O. Seely, Paola P. Pagan, Prem R. Warde, Labu J. Tamrakar, Dipen J. Parekh, Tanira Ferreira, Yi Zhou, Hayley B. Gershengorn, Bhavarth S. Shukla

**Affiliations:** 1 University of Miami Health System, Miami, FL, USA; 2 Department of Biochemistry and Molecular Biology, University of Miami Miller School of Medicine, Miami, FL, USA; 3 Division of Infectious Diseases, Department of Internal Medicine, University of Miami Miller School of Medicine, Miami, FL, USA; 4 Department of Urology, University of Miami Miller School of Medicine, Miami, FL, USA; 5 Department of Medicine, Division of Pulmonary, Critical Care, and Sleep Medicine, University of Miami Miller School of Medicine, Miami, FL, USA; 6 Department of Pathology and Laboratory Medicine, University of Miami Miller School of Medicine, Miami, FL, USA; 7 Department of Medicine, Division of Critical Care, Albert Einstein College of Medicine, Bronx, NY, USA

## Abstract

**Objective:**

Assess turnaround time (TAT) and cost-benefit of on-site *C. auris* screening and its impact on length of stay (LOS) and costs compared to reference laboratories.

**Design:**

Before-and-after retrospective cohort study.

**Setting:**

Large-tertiary medical center.

**Methods:**

We validated an on-site polymerase chain reaction-based testing platform for *C. auris* and retrospectively reviewed hospitalized adults who screened negative before and after platform implementation. We constructed multivariable models to assess the association of screening negative with hospital LOS/cost in the pre and postimplementation periods. We adjusted for confounders such as demographics and indwelling device use, and compared TATs for all samples tested.

**Results:**

The sensitivity and specificity of the testing platform were 100% and 98.11%, respectively, compared to send-out testing. The clinical cohort included 287 adults in the pre and 1,266 postimplementation period. The TAT was reduced by more than 2 days (3 (interquartile range (IQR): 2.0, 7.0) vs 0.42 (IQR: 0.24, 0.81), *p* < 0.001). Median LOS was significantly lower in the postimplementation period; however, this was no longer evident after adjustment. In relation to total cost, the time period had an effect of $6,965 (95% CI: −$481, $14,412); *p* = 0.067) on reducing the cost. The median adjusted total cost per patient was $7,045 (IQR: $3,805, $13,924) less in the post vs the preimplementation period.

**Conclusions:**

Our assessment did not find a statistically significant change in LOS, nevertheless, on-site testing was not cost-prohibitive for the institution. The value of on-site testing may be supported if an institutional *C. auris* reduction strategy emphasizes faster TATs.

## Background

Since *Candida auris* was first identified in 2009,^1^ documented cases have spread worldwide across 49 nations in every continent except Antarctica.^2^ It is suggested that the actual spread of *C. auris* is far greater than what is currently understood, likely due to difficulties with detection capacity.^3^ In addition, its ability to colonize patients’ skin and survive on abiotic surfaces for weeks contributes to the proliferation of *C. auris* in healthcare facilities.^4^ These challenges have been documented to cause outbreaks throughout the world, which were only exacerbated by the COVID-19 pandemic.^5–13^ In fact, acute-care settings experienced increased *C. auris* transmission during the pandemic, especially in COVID-19 units.^12,13^ Furthermore, surveillance efforts, particularly colonization screening, were shifted toward pandemic response, which likely resulted in silent amplification of *C. auris*.^12,13^ This makes the detection of *C. auris* a necessary step to contain it as its absence could cause delayed infection prevention and control (IPC) interventions.^14^

Additionally, a 2020 study noted how reliance on biochemical-based identification systems, such as Vitek-2, was challenging since *C. auris* was not present in their databases.^15^ Another study, in 2021, noted that lack of on-site PCR-testing capacity for *C. auris* is a limitation for surveillance in the U.S.^16^ The Centers for Disease Control and Prevention (CDC) recommends screening an index patient’s contacts when a new case is identified.^17^ However, as on-site PCR testing is not widely available in the US,^12,15,16^ facilities may send out samples to public health or reference laboratories, increasing turnaround time (TAT) to multiple days.^16^

Moreover, extended TAT can cause difficulties such as transfer delays to long-term care facilities (LTCFs), strain healthcare resources, and increase risks for transmission.^16^ Multidrug-resistant organisms have long been associated with increased costs compared to susceptible strains, and *C. auris* is no exception.^18,19^ Here, we evaluate whether our increase in testing capacity was associated with better TAT, shorter hospital lengths of stay (LOSs), and lower costs.

## Methods

We conducted a retrospective cohort study of adults hospitalized at a tertiary academic medical center in Miami, FL who screened negative for *C. auris* colonization and assessed TAT of all screening tests conducted (positive and negative). Prior to June 18, 2021, all screening tests were sent out to be performed by the CDC’s Antimicrobial Resistance Laboratory Network (ARLN). On June 18, 2021, our institution implemented on-site polymerase chain reaction (PCR) testing for *C. auris* colonization.

### On-site laboratory validation of C. auris testing platform

Analytical validation of the BioGX *C. auris* Open System Reagents for BD MaxTM (REF 350-070-C-MAX) was performed by establishing limit of detection, reproducibility, and analytical specificity. The limit of detection (LoD) was determined using the BioGX *C. auris* BD positive control and was reconstituted to contain 5,000 genomic copies/µL. A ten-fold serial dilution from 5,000 copies/µL to 0.1 copies/µL was tested in triplicate. Between runs, accuracy was determined using 10 positive (105 copies/µL of control DNA) and 10 negative samples that were tested on different days and using different operators.

The impact of factors that may interfere with the PCR reaction was also evaluated. The four potential interfering substances we tested were sweat, soap/detergent, 100% ethanol, and blood. *C. auris* control DNA was diluted to 3 copies/µL in BD ESwab modified liquid Amies medium and inoculated with trace amounts (2–4 µL) of interfering substances and tested in duplicate. Storage temperature was also evaluated as a potential interfering factor. Positive controls (3 copies/µL) were stored at 4–8°C or room temperature for 24 and 48 hours. The samples were then tested in duplicate. Cross-reactivity with non-related pathogens was tested in silico using Nucleotide Basic Local Alignment Search Tool (BLASTn) (NCBI),^20^ and in vitro testing of similar pathogens (*C. duobushaemulonii*, *C. haemulonii*, *S. cerevisiae*, *C. krusei*, *K. ohmeri*, and *C. lusitaniae*, acquired from CDC) was determined using sample isolates inoculated in ESwab media.

Clinical validation of the BioGx *C. auris* BD Max test was performed using clinical specimens: composite axilla/groin samples were collected in duplicate in BD ESwab modified liquid Amies medium. One batch of duplicate samples was sent to ARLN and the other was tested using the BD MAX. Sensitivity and specificity of the on-site platform were assessed using ARLN results as the reference PCR standard.

### Comparison of test turnaround time

We compared the change in TAT between the pre and postimplementation period by gathering the collection date of each PCR test and the reported date of each test result. Report and collection dates for the preimplementation period were assembled using ARLN requisition spreadsheets and our electronic health record was used for the postimplementation period. Wilcox rank sum testing was used to compare TAT between the pre and postimplementation periods. Our evaluation included all composite axilla/groin skin colonization tests (admission screenings and point prevalence surveys [PPS]), positive and negative, performed on patients with risk factors for *C. auris* and those who were screened for discharge purposes as requested by postacute care facilities. Our definition of high-risk varied over time due to external factors such as COVID-19 case surges, local transmission patterns, and CDC guidelines; but primarily, ARLN testing capacity and availability, and on-site PCR testing are what dictated our screening program in the pre and postimplementation period, respectively. For example, the risk factors that prompted *C. auris* PPSs included exposure to newly identified in-house *C. auris*-positive patients, such as overlap in time and space within the same ward. Factors that prompted admission screening were transfers from LTCFs with known transmission as reported by the local health department, and/or transfer patients on mechanical ventilation and/or with tracheostomies. These risk factors were then evaluated against the current ARLN testing capacity to assess how broad or narrow our PPSs needed to be.

### Clinical study cohort

We included all hospitalized adults who screened negative with an axilla/groin composite swab for *C. auris* between July 1, 2020, and March 31, 2022, to evaluate LOS and cost-saving. Patients who were deemed high-risk for *C. auris* colonization were screened on admission to the hospital. All patients requiring discharge to an LTCF were screened during discharge planning if requested by the receiving facility. We focused on patients who screened negative for *C. auris* as this is the subgroup for whom discharge may be needlessly delayed by longer testing TAT. We presumed that patients who screened positive were likely to have excess LOS related to *C. auris* colonization itself rather than testing delays. Patients were excluded from this cohort if they tested positive for *C. auris* or did not require screening.

### Exposure and outcomes

A negative screen for *C. auris* during the pre (July 1, 2020–June 17, 2021) and postperiod (June 18, 2021–March 31, 2022) rendered patients eligible for inclusion into the cohort. Patients who screened negative by in-house testing were compared with those who screened negative by send-out testing. Our primary clinical outcome was hospital LOS. We secondarily evaluated expected cost-savings associated with on-site test implementation. Although we did not complete a formal survey, we solicited qualitative feedback regarding the impact on clinical operations from hospital leadership as well as team leads for nursing, environmental services, bed placement, social work, and the emergency department.

### Statistical analyses

We used counts with percentages and medians with interquartile ranges (IQRs) to describe cohort characteristics. Chi-square and Wilcox rank sum testing were used, as appropriate, to compare characteristics between patients admitted in the pre to postimplementation periods. We constructed a multivariable linear regression model to assess the association of screening negative for *C. auris* during the postimplementation period (vs the preimplementation period) with hospital LOS after adjustment for a priori determined potential confounders: age, gender, race/ethnicity, primary insurance provider, chronic health conditions (number of Elixhauser comorbidities),^21^ need for invasive mechanical ventilation in the year prior to hospital admission, prior history of a tracheostomy tube, severity of acute illness, arrival from a skilled nursing facility, maximum sequential organ failure assessment (SOFA) score within 24 hours of hospital admission,^22^ receipt of a central venous catheter during hospitalization, receipt of a urinary catheter during hospitalization, positive COVID-19 PCR result or COVID-19 diagnosis code during hospitalization, and discharge disposition. We logarithmically transformed hospital LOS for use as the dependent variable to improve normality; as such, model regression coefficients represent a percent change.

We considered the potential cost-savings of on-site testing by balancing the costs of the program with potential costs saved by reductions in LOS associated with avoiding TAT delays. The cost per test includes supplies (Biogx reagent, strip BD, and miscellaneous supplies such as specimen container and tips), equipment (BD service contract), full-time equivalent (FTE) (including collection labor), and a 20% overhead cost for administrative items. To consider potential hospitalization-associated cost reductions, we obtained total hospital charges for each cohort patient (pre and postimplementation). All charges were converted to 2020 dollars using the US Bureau of Labor Statistics’ consumer price index for hospital services from December 2020 to December 2021.^23^ We then used multivariable linear regression modeling (dependent variable: charges in 2020 dollars; independent variables: same as for LOS model above) to calculate adjusted charges for each patient. The median, 25th percentile, and 75th percentile of adjusted charges before and after on-site testing initiation were determined; the adjusted charges avoided by on-site screening were then calculated as the difference in the medians (range defined by the difference in the 25th and 75th percentiles) between patients screened after and before on-site testing. Finally, charges were converted to costs using the Centers for Medicare & Medicaid Services 2020 cost-to-charge ratios (operating plus capital ratios) for Florida urban acute care hospitals.^24^ The total differential cost between the pre and posttime period was then found.

All analyses were performed using R (R Foundation for Statistical Computing, Vienna, Austria) and Excel (Microsoft Office 365, Redmond, WA). This project was approved by the University of Miami Institutional Review Board (#20210224).

## Results

### Platform validation

The lowest consistently detected concentration of *C. auris* DNA was 1 copy/µL and the LoD was repeated 16 times with an overall agreement of 100% with cycle threshold (Ct) value coefficient of variation (CV) being 3.036%. The inter-run CV of the Ct values was determined as 2.651%. Out of the four potential interfering substances, only high concentrations of ethanol significantly affected the Ct value. Testing performed at different temperatures (4–8°C and room temperature) revealed results with 100% agreement. Cross-reactivity testing of non-related pathogens did not show any significant similarity (>90%) and testing with similar pathogens detected all organisms except *C. auris*. Finally, the sensitivity and specificity of the 69 duplicate samples were determined as 100% (16/16 ALRN positive samples; [95% CI: 79.9, 100%]) and 98.11% (52/53 ALRN negative samples; [95% CI: 89.93, 99%]), respectively.

### Operational impact

**Figure 1.**
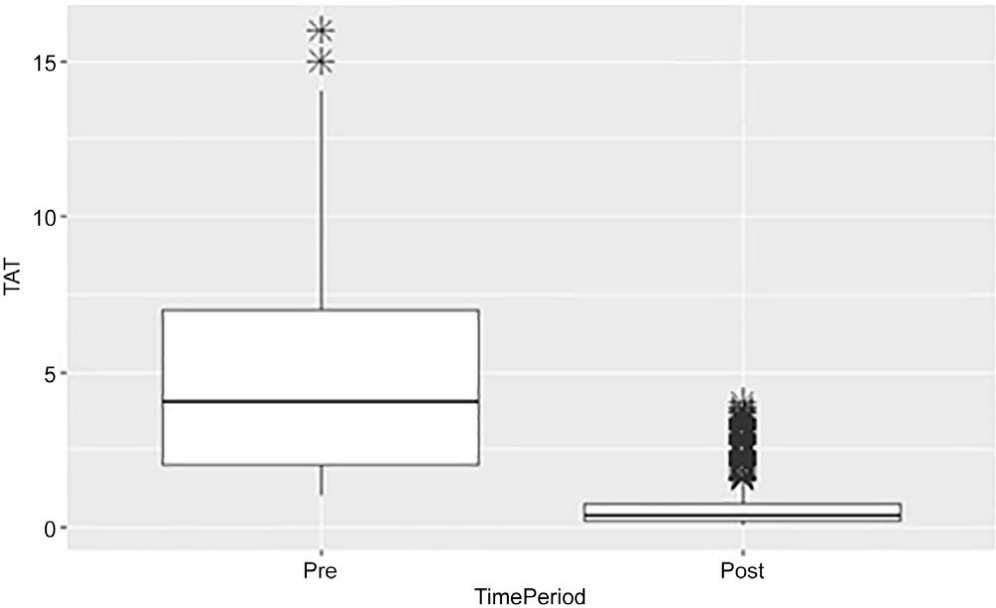
Comparison of testing turnaround time between time periods for all tests.

For the TAT comparison, we evaluated all tests regardless of result with a total of 2,067 test results during the pre (*N* = 632) and postimplementation periods (*N* = 1,435). The TAT was reduced in the postimplementation period by more than 2 days (3 [IQR: 2.0, 7.0] vs 0.42 [IQR: 0.24, 0.81], *p* < 0.001) (Figure 1, Table 1).

**Table 1.** Number of tests assessed to compare TAT

	Overall	Preimplementation period	Postimplementation period	*p*-value
*N* (%)	2,067	632	1,435	
TAT, median (IQR)	0.76 (0.31,2.0)	3.0 (2.0, 7.0)	0.42 (0.24, 0.81)	<0.001

IQR, interquartile range.

Our primary clinical cohort consisted of 1,553 patients who screened negative for *C. auris*: 287 (18.5%) were part of the preimplementation period cohort and 1,266 (81.5%) belonged to the postimplementation period cohort (Table 2). Patient demographics did not differ between periods, such as age (72 [IRQ: 60, 81] vs 70 [IQR: 57, 81], *p* = 0.067); however, some clinical factors such as mechanical ventilation usage during hospitalization were significantly different between periods (208 [16%] vs 69 [24%], *p* = 0.004). Additionally, postimplementation patients were less chronically ill (Elixhauser comorbidity index: 7.0 [IQR: 5.0, 10.0] vs 9.0 [IQR: 6.0, 11.0], p < 0.001) and required fewer resources during hospitalization (central line: 34 [2.7%] vs 87 [30%], *p* < 0.001; urinary catheter: 448 [35%] vs 126 [44%], *p* = 0.008).

**Table 2.** Negative screening test cohort characteristics stratified by time period

Characteristic	Overall	Preimplementation period	Postimplementation period	p-value
**N, row %**	1,553	287 (18.5%)	1,266 (81.5%)	
**Length of stay,** days	9 (5, 17)	11 (6, 22)	8 (5, 16)	<0.001
**Age,** years	70 (58, 81)	72 (60, 81)	70 (57, 81)	0.067
**Gender,** male	809 (52%)	168 (59%)	641 (51%)	0.016
**Payer**				0.6
Commercial	288 (19%)	46 (16%)	242 (19%)	
Government	37 (2.4%)	7 (2.4%)	30 (2.4%)	
Medicaid	191 (12%)	34 (12%)	157 (12%)	
Medicare	996 (64%)	190 (66%)	806 (64%)	
Other	41 (2.6%)	10 (3.5%)	31 (2.4%)	
**Race/Ethnicity**				0.001
White Non-Hispanic	248 (16%)	57 (20%)	191 (15%)	
White Hispanic	848 (55%)	144 (50%)	704 (56%)	
Black Non-Hispanic	294 (19%)	41 (14%)	253 (20%)	
Black Hispanic	35 (2.3%)	8 (2.8%)	27 (2.1%)	
Other	128 (8.2%)	37 (13%)	91 (7.2%)	
**Central line usage**	121 (7.8%)	87 (30%)	34 (2.7%)	<0.001
**Urinary catheter usage**	574 (37%)	126 (44%)	448 (35%)	0.008
**Elixhauser Comorbidity Index**	8.0 (5.0, 10.0)	9.0 (6.0, 11.0)	7.0 (5.0, 10.0)	<0.001
**Received mechanical ventilation during hospitalization**	277 (18%)	69 (24%)	208 (16%)	0.004
**Received mechanical ventilation during any hospitalization in the previous year**	103 (6.6%)	35 (12%)	68 (5.4%)	<0.001
**History of tracheostomy usage**	27 (1.7%)	11 (3.8%)	16 (1.3%)	0.009
**Disposition**				<0.001
Expired	158 (10%)	37 (13%)	121 (9.6%)	
Facility	537 (35%)	133 (46%)	404 (32%)	
Home	830 (53%)	113 (39%)	717 (57%)	
Other	28 (1.8%)	4 (1.4%)	24 (1.9%)	
**COVID**	751 (48%)	108 (38%)	643 (51%)	<0.001
**PCP on file**	685 (44%)	144 (50%)	541 (43%)	0.025
**BMI**	27 (23, 32)	27 (23, 32)	27 (23, 32)	>0.9
**Cancer diagnosis**	696 (45%)	151 (53%)	545 (43%)	0.004
**Max SOFA score within 24 hours of admission**	3.00 (1.00, 5.00)	3.00 (2.00, 5.00)	2.00 (1.00, 5.00)	<0.001
**Arrival from skilled nursing facility**	294 (19%)	73 (25%)	221 (17%)	0.003
Elixhauser comorbidities*				
AIDS	40 (2.6%)	5 (1.7%)	35 (2.8%)	0.4
Alcohol abuse	72 (4.6%)	15 (5.2%)	57 (4.5%)	0.6
Deficiency anemia	949 (61%)	196 (68%)	753 (59%)	0.006
Rheumatoid arthritis/CVD	94 (6.1%)	19 (6.6%)	75 (5.9%)	0.7
Blood loss anemia	81 (5.2%)	27 (9.4%)	54 (4.3%)	0.001
Congestive heart failure	524 (34%)	105 (37%)	419 (33%)	0.3
Chronic pulmonary dis.	534 (34%)	115 (40%)	419 (33%)	0.028
Coagulopathy	426 (27%)	87 (30%)	339 (27%)	0.2
Depression	490 (32%)	108 (38%)	382 (30%)	0.017
Diabetes mellitus				
Uncomplicated	541 (35%)	110 (38%)	431 (34%)	0.2
Complicated	638 (41%)	126 (44%)	512 (40%)	0.3
Drug abuse	82 (5.3%)	11 (3.8%)	71 (5.6%)	0.3
Hypertension	1,284 (83%)	256 (89%)	1,028 (81%)	<0.001
Hypothyroidism	348 (22%)	91 (32%)	257 (20%)	<0.001
Liver disease	248 (16%)	51 (18%)	197 (16%)	0.4
Lymphoma	90 (5.8%)	19 (6.6%)	71 (5.6%)	0.5
Fluid and electrolyte dis.	1,186 (76%)	237 (83%)	949 (75%)	0.006
Metastatic cancer	199 (13%)	44 (15%)	155 (12%)	0.2
Other neurologic dis.	520 (33%)	126 (44%)	394 (31%)	<0.001
Obesity	651 (42%)	120 (42%)	531 (42%)	>0.9
Paralysis	210 (14%)	59 (21%)	151 (12%)	<0.001
Peripheral vascular dis.	420 (27%)	92 (32%)	328 (26%)	0.039
Psychoses	153 (9.9%)	40 (14%)	113 (8.9%)	0.015
Pulmonary circulation dis.	293 (19%)	53 (18%)	240 (19%)	>0.9
Renal failure	582 (37%)	123 (43%)	459 (36%)	0.043
Solid tumor without metastasis	321 (21%)	74 (26%)	247 (20%)	0.019
Peptic ulcer disease	97 (6.2%)	21 (7.3%)	76 (6.0%)	0.4
Valvular disease	340 (22%)	62 (22%)	278 (22%)	>0.9
Weight loss	479 (31%)	103 (36%)	376 (30%)	0.047

IQR, interquartile range; med, median; SOFA, sequential organ failure assessment; BMI, body mass index; PCP, primary care physician; Dis., Disorder; CVD, collagen vascular diseases.

* There was one case with unknown Elixhauser comorbidities.

The unadjusted median LOS was lower in the post vs the preperiod (8 d [IQR: 5, 16] vs × 11 d [IQR: 6, 22]; (34% reduction [IQR: −48%, −21%], *p* < 0.001; Tables 2 & 3). After adjusting for confounders, we identified an 8% reduction ([IQR: −21%, 5%], *p* = 0.20) in LOS in the post vs the preimplementation period testing, but this reduction was not significant.

**Table 3.** Unadjusted and adjusted models for length of stay for the negative screening test cohort

Characteristic	Unadjusted			Adjusted		
	% Change	95% CI^1^	*p*-value	% Change	95% CI^1^	*p*-value
**Time period**			**<0.001**			0.2
Pre on-site testing	—	—		—	—	
Post on-site testing	−34%	−48%, −21%		−8%	−21%, 5%	
**Age,** years	0%	0%, 0%	0.7	0%	−1%, 0%	0.1
**Gender,** male	7%	−3%, 18%	0.2	5%	−4%, 14%	0.3
**Race/Ethnicity**		0%, 0%	0.6		0%, 0%	0.4
White Non-Hispanic	—	—		—	—	
Black Hispanic	-23%	−60%, 14%		−22%	−55%, 10%	
Black Non-Hispanic	8%	−10%, 25%		7%	−9%, 23%	
Other	4%	−18%, 27%		2%	−17%, 22%	
White Hispanic	3%	−12%, 18%		5%	−8%, 18%	
**Payer**		0%, 0%	**0.021**		0%, 0%	0.3
Commercial	—	—		—	—	
Government	−1%	−37%, 35%		16%	−15%, 47%	
Medicaid	11%	−8%, 30%		8%	−9%, 25%	
Medicare	4%	−10%, 18%		−5%	−20%, 10%	
Other	−49%	−83%, −15%		−16%	−46%, 14%	
**SOFA**	6%	4%, 8%	**<0.001**	1%	−1%, 3%	0.4
**Received mechanical ventilation during any hospitalization in the previous year**	97%	77%, 120%	**<0.001**		0%, 0%	
**History of tracheostomy usage**	110%	72%, 150%	**<0.001**	84%	49%, 120%	**<0.001**
**Central line usage**	88%	69%, 110%	**<0.001**	45%	26%, 64%	**<0.001**
**Urinary catheter usage**	70%	60%, 80%	**<0.001**	64%	54%, 75%	**<0.001**
**COVID**	14%	4%, 25%	**0.008**	45%	35%, 56%	**<0.001**
**Disposition**		0%, 0%	**<0.001**		0%, 0%	**<0.001**
Expired	—	—		—	—	
Facility	19%	1%, 38%		53%	36%, 70%	
Home	−31%	−48%, −13%		9%	−8%, 26%	
Other	−55%	−96%, −14%		−26%	−64%, 12%	
**Elixhauser Comorbidity Index**	6%	5%, 8%	**<0.001**	7%	5%, 8%	**<0.001**
**Arrival from skilled nursing facility**	−14%	−27%, −1%	**0.036**	−21%	−34%, −8%	**0.002**

The multivariable linear regression for charges showed that time period was not statistically significant: time period had an effect of $6,965 ([95% CI: −481, 14,412], *p* = 0.067) on total charges. However, the results were still used to assess the differences in cost between periods. The adjusted median hospitalization-associated cost-savings per patient were predicted to be $7,045 (IQR: $3,805, $13,924) in the postimplementation period (Table 4). The price for each *C. auris* PCR test was $63.72 in the postimplementation period, which was inclusive of labor and the validation costs for the equipment. Based on the average number of tests per patient in the postperiod (1.17), the total potential cost-savings inclusive of operational cost per patient was $6,970 (IQR: $3,730, $13,849).

**Table 4.** Costs associated with on-site testing program

	Pre	Post	Difference (Pre-Post)
Cost per test	--	63.72	(63.72)
Cost per patient*, median (IQR)	31,541 (20,317, 55,585)	24,496 (16,512, 41,661)	7,045 (3,805, 13,924)
Total Cost, median (IQR)	31,541 (20,317, 55,585)	24,571 (16,587, 41,736)	6,970 (3,730, 13,849)

IQR, interquartile range.

* Cost per patient is the adjusted cost from the multivariable regression model.

Informal feedback obtained during the postimplementation period was overwhelmingly positive. Bed placement and nursing leaders reported increased efficiency with placement of patients and use of isolation. Among leadership, there was a change in perspective to PPSs, where they were now seen as helpful instead of a strain on operations. Social work leaders reported a decrease in barriers to discharge planning as colonization status was now identified in a more timely manner.

## Conclusions

*C. auris* poses operational challenges for healthcare facilities. Thus, with the support of hospital leadership, we invested in the validation of an on-site testing platform, which yielded excellent validity. While this platform did not result in a significant change in LOS, it was not cost-prohibitive for the institution. We did find a reduction of TATs associated with its implementation and positive feedback from hospital staff.

We expected the shortened TAT with in-hospital testing to be associated with a reduction in LOS. Although the unadjusted LOS was shorter following implementation, after adjustment, this reduction was no longer significant. The case mix of patients screened for *C. auris* was notably different in the two time periods. This difference stemmed from our being more selective on whom to screen in the pre (*N* = 632) vs the postimplementation period (*N* = 1435) due to limited ALRN testing, especially admission screening, which required additional approval. Increased access to testing and shorter TAT expanded the amount of screening performed, specifically with regard to admission screenings and PPSs. It is likely that these postperiod patients were different in unmeasured ways from those tested in the preperiod and, thus, residual confounding may impact our findings.

On-site testing capacity did afford us a reduction in TAT to fewer than 9 hours from multiple days. It also negated the need for a dedicated infection preventionist to be involved in coordinating screening testing via the ARLN. Most notably though, the expanded testing permitted us to designate a ward to cohort *C. auris*-positive and “rule-out” patients. Patients with risk factors were cohorted immediately upon admission or when tested during a PPS and the reduced TAT allowed us to quickly transfer them out of the *C. auris* cohort unit if they tested negative. This allowed us to decrease transmission risks between “rule-out” patients and *C. auris*-positive patients by decreasing the time they overlapped in time and space. For the isolation ward, we were able to designate dedicated staff, equipment, and special education for staff. Furthermore, for patients requiring specialty care in the *C. auris* ward, we were able to assign specialized nurses from other wards to care for the patient without transferring *C. auris*-positive patients out of the *C. auris* cohort ward. In this manner, we were able to isolate *C. auris*-positive patients to one specific area of the hospital, and thereby reduce transmission risks to other patients. Additionally, we were able to refine our admission screening protocol and modify it to our local needs. This allowed us to target specific risk factors based on local transmission trends, such as screening from specific LTCFs, instead of only relying on general CDC guidance. It is possible that with a larger sample size, we may have seen these interventions result in a significant reduction in LOS.

To our knowledge, this is the only publication to assess the impact of an in-house *C. auris* PCR-testing platform on hospital operations. Nucleic acid amplification techniques for *C. auris* have been developed in several platforms since 2014 but most continue to be inaccessible for frontline laboratories, LTCFs, and acute-care hospitals.^15,16,25^ Consequently, this type of intervention is well-suited for other acute-care hospitals and postacute care facilities that care for high-risk patients and can invest in a *C. auris* on-site testing platform. This would relieve the burden from public health laboratories (e.g., ARLN) and increase testing availability for communities nationwide.

Our study has several limitations. First, the case mix of patients in the pre and postimplementation periods differed across measured characteristics; how and if they differed in unmeasured ways and to what extent these differences residually confound our results is unknown. Second, data on admission, discharge, or PPS screening per patient were not consistently described for negative results. Specifically, we were not able to identify the indication for testing in each case. Third, we did not collect data on the impact of isolation requirements for patients under investigation for *C. auris* colonization, which is, possibly, an important impact of our in-house testing implementation. Additionally, while our sample included 1,553 patient encounters and 2,067 tests, only one facility was studied in our analysis, which could limit the generalizability of our results. Finally, a detailed analysis of bed days saved from the *C. auris* unit implementation may have provided further insight into operational impact of the on-site test; however, we did not have bed mapping designation based on *C. auris* “rule-out”/positivity in the electronic health record to conduct this analysis.

In conclusion, the detection of *C. auris* is imperative to its control. Many facilities currently lack the capacity and leadership support to screen for and rapidly identify colonization of the organism by using an in-house platform, thus creating the perfect environment for silent amplification. Our findings suggest that there is value in investing in the expansion of more rapid onsite testing capability. This has the potential to not only save resources but also to enhance targeted IPC practices and to prevent further transmission events by identifying colonized patients more rapidly.
